# Unrecognized Motor Difficulties and Developmental Coordination Disorder in Preschool Children

**DOI:** 10.1001/jamanetworkopen.2025.36227

**Published:** 2025-10-07

**Authors:** Barbara Scheiber, Claudia Spiegl, Jasmin Plattner, Sarah Mildner, Peter Federolf

**Affiliations:** 1 Department of Physiotherapy, University of Applied Sciences Tyrol, Innsbruck, Austria; 2 Department of Sport Science, University of Innsbruck, Innsbruck, Austria

## Abstract

This cross-sectional study assesses the prevalence of motor difficulties suggestive of developmental coordination disorder among children and whether parents are aware of these difficulties.

## Introduction

Developmental coordination disorder (DCD) affects 5% to 6% of school-aged children, impairing motor function, daily activities, and academic performance.^1^ While diagnosis typically occurs once children are in school, motor deficits often manifest earlier, yet current early-childhood welfare systems seem not fully equipped for identifying DCD.^2,3^ Without timely support, children with DCD are at high risk of secondary complications.^4^ This study introduces a 2-stage screening approach using validated clinical tools to address the prevalence of motor difficulties suggestive of DCD among preschool-aged children and whether parents are aware of these difficulties.

## Methods

This cross-sectional, 2-stage screening study was conducted between September 23 and October 25, 2024, in 25 kindergartens representing the preschool population of Tyrol, Austria (eTable in Supplement 1). Ethics approval was obtained from the Research Committee for Scientific Ethical Questions and the Ethical Board of the University of Innsbruck. Parental awareness questionnaires (eAppendix in Supplement 1) and consent forms were distributed via kindergartens. This study followed the STROBE reporting guideline.

In stage 1, children were screened using the MobiScreen 4-6, a standardized and validated motor test battery specifically designed for preschoolers and that meets clinical standards.^5^ In stage 2, children slower than the test-specific cutoff for their age group participated in a detailed individual assessment using the Movement Assessment Battery for Children-2,^6^ an internationally recognized criterion standard for diagnosing DCD (scoring classifications in the Table).

**Table.** Presentation of Results for the MobiScreen 4-6 and M-ABC-2

Variable	MobiScreen 4-6 categorization^a^		M-ABC-2 group^b^					
	Inconspicuous	Conspicuous	Manual dexterity 1	Aiming and catching 1	Balance 1	Manual dexterity 2	Aiming and catching 2	Balance 2
No. of participants (%)	568 (75.3)	186 (24.7)	60 (32.3)	86 (46.2)	87 (46.8)	43 (23.1)	16 (8.6)	14 (7.5)
Time, median (IQR), s	24 (21-27)	36 (31-43)	NA	NA	NA	NA	NA	NA
Score, median (IQR)^c^	23 (20-24)	22 (20-24)	37 (16-63)	50 (37-72)	63 (37-91)	5 (1-9)	5 (2-6)	9 (6-9)

Abbreviations: M-ABC-2, Movement Assessment Battery for Children, Second Edition; NA, not applicable.

^a^ MobiScreen 4-6 categorization is based on the age-related cutoff values (conspicuous results for 4 years, ≥39 seconds; 5 years, ≥29 seconds; 6 years, ≥26 seconds).

^b^ Children who scored at or below the 5th percentile on the M-ABC-2 were classified as in need of therapy, indicating substantial difficulties in the respective dimension of the M-ABC-2 (manual dexterity, aiming and catching, balance). Those scoring between the 6th and 15th percentiles were classified as at risk or within the borderline range, indicating a critical level of motor coordination difficulties that warrants monitoring or further evaluation. Group 1 scoring greater than 15th percentile; group 2 scoring less than or equal to 15th percentile.

^c^ The MobiScreen 4-6 consists of 5 stations with different tasks. Each task is scored on a scale of 0 to 5 points for a maximum score of 25 points, which is the best possible outcome. For the MobiScreen 4-6, scores are only relevant for possible future planning of interventions, while the time cutoff values are used to determine classification in conspicuous or inconspicuous.

All children aged 4 to 6 years enrolled in a participating kindergarten were eligible unless unable to complete the assessment due to severe cognitive or physical impairment as determined by kindergarten staff. Assessments were conducted by 5 teams of 2 physiotherapists each.

Descriptive statistics were use to summarize participant characteristics (age, sex, body mass index [BMI, calculated as weight in kilograms divided by height in meters squared]) and screening outcomes, and percentages were calculated for prevalence rates and response patterns. The BMI was assessed to see whether overweight children would have worse results. The data were analyzed using Jamovi, version 2.6 (Jamovi Project).

## Results

Of 754 participating children (median [range] age, 5 [4-6] years, 378 female [50.1%] and 376 male [49.9%]), the median BMI was 15.2 (IQR, 14.2-16.2). The parental response rate for completed questionnaires was 66.6%. All included children were screened in stage 1, of whom 186 (24.7%) (median [IQR] BMI, 15.3 [14.4-16.4]) were referred to stage 2 (Table; Figure). Stage 2 assessments identified 33 children (4.4%) needing therapy and 16 children (2.1%) with critical motor skills. Notably, none of these children’s parents had expressed concerns on the questionnaires. Overall, 717 parents (95.1%) reported no motor concerns, 28 (3.7%) noted past issues, and 9 (1.2%) gave no information. Previously reported motor difficulties in 28 children were not further tracked as the issues were no longer evident at the time of assessment. Only 12 parents (1.6%) stated that concerns persisted, but only 1 child advanced to stage 2, in which typical motor development was observed.

**Figure.**
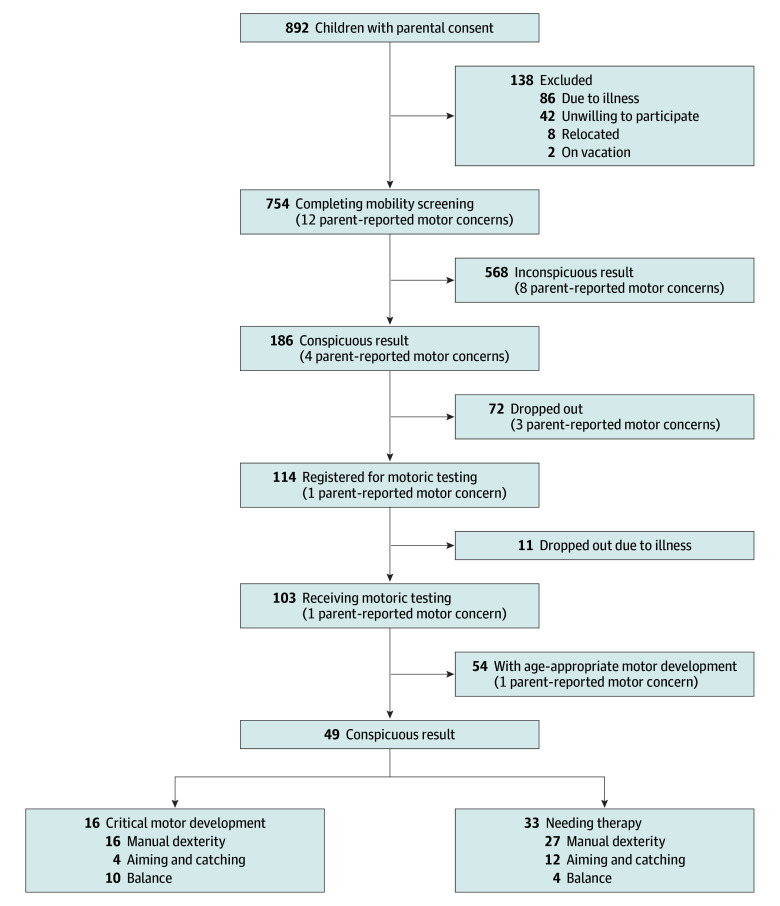
Flowchart of the 2-Stage Screening Process Conspicuous results are based on MobiScreen 4-6 categorization of age-related cutoff values (4 years, ≥39 seconds; 5 years, ≥29 seconds; 6 years, ≥26 seconds).^5^ Children who scored at or below the 5th percentile on the Movement Assessment Battery for Children, Second Edition (M-ABC-2) were classified as in need of therapy, indicating substantial difficulties in the respective dimension of the M-ABC-2 (manual dexterity, aiming and catching, balance).^6^

## Discussion

This cross-sectional study aligns with the reported prevalence of DCD and reinforces concerns about underrecognition. None of the parents of children with substantial motor difficulties reported awareness, underscoring the value of structured, objective screening. Unlike earlier studies that focused on older children,^2^ ours offers a systematic, scalable approach for early identification in preschoolers that supports timely referral to clinicians for formal diagnosis.

The 39% attrition rate reflects challenges in longitudinal pediatric assessments and the necessity of implementing screenings into kindergarten settings. Parental reports highlighted a critical point: Even when asked directly, parents often do not recognize motor difficulties in their children. Study limitations include a lack of data on nonparticipants, limiting comparison; attrition between stages, which may have influenced prevalence estimates; and absence of formal DCD diagnoses, despite using validated tools.

Our study shows that many preschoolers with motor deficits remain undetected in current preventive checkups. Two-stage screening offers a practical solution for early detection and may improve access to intervention and long-term outcomes for children with DCD.

## Notes

Supplement 1. **eTable.** Presentation of Response Rates and Selection of Kindergartens Based on the Population Figures of the Nine Tyrolean Districts **eAppendix.** Parent Questionnaire–Mobility Screening Participation

Supplement 2.
Data Sharing Statement
