# Supplementary material for: Unrecognized Motor Difficulties and Developmental Coordination Disorder in Preschool Children
Source: JAMA Netw Open. 2025 Oct 7;8(10):e2536227. doi: 10.1001/jamanetworkopen.2025.36227 (PMC12505165; doi:10.1001/jamanetworkopen.2025.36227)
Supplement: Supplement 1. — eTable. Presentation of Response Rates and Selection of Kindergartens Based on the Population Figures of the Nine Tyrolean Districts eAppendix. Parent Questionnaire–Mobility Screening Participation [file jamanetwopen-e2536227-s001.pdf]

Supplemental Online Content

Scheiber B, Spiegl C, Plattner J, Mildner S, Federolf P. Unrecognized motor difficulties and developmental coordination disorder in preschool children. *JAMA Netw Open*. 2025;8(10):e2536227. doi:10.1001/jamanetworkopen.2025.36227

eTable. Presentation of Response Rates and Selection of Kindergartens Based on the Population Figures of the Nine Tyrolean Districts

eAppendix. Parent Questionnaire–Mobility Screening Participation

This supplemental material has been provided by the authors to give readers additional information about their work.

© 2025 Scheiber B et al. *JAMA Network Open*.

eTable. Presentation of Response Rates and Selection of Kindergartens Based on the Population Figures of the Nine Tyrolean Districts

District	Population (n)	KG per district (n)	RR KG consent (n (%))	Selected KG (n)	Eligible children (n)	RR Parental consent (n (%))
Innsbruck-Land	186,121	105	16 (15.2)	6	328	199 (60.7)
Innsbruck-Stadt	132,174	62	4 (6.5)	4	159	87 (54.7)
Kufstein	114,013	63	15 (23.8)	4	258	190 (73.6)
Schwaz	87,187	62	10 (16.1)	3	187	132 (70.6)
Kitzbühel	66,085	27	6 (22.2)	2	125	106 (84.8)
Imst	62,740	46	8 (17.4)	2	132	78 (59.1)
Lienz	48,847	45	3 (6.7)	2	62	53 (87.1)
Landeck	45,065	42	7 (16.7)	1	64	27 (42.2)
Reutte	33,850	34	5 (14.7)	1	25	20 (80.0)
**Total**	**776,082**	**486**	**72 (14.8)**	**25**[a]	**1,340**	**892 (66.6)**[b]

Abbreviations and footnotes: KG Kindergarten; RR response rate; n number; % percent; [a] 28% urban, 72% rural; [b] 62.2% urban, 67.8% rural

© 2025 Scheiber B et al. *JAMA Network Open*.

eAppendix. Parent Questionnaire–Mobility Screening Participation

1. First and last name of your child:

2. Date of birth of your child: ____ / ____ / ______ (DD.MM.YYYY)

3. Gender of your child:

☐ Female ☐ Male ☐ Prefer not to say ☐ Other: _____________

4. Since when has your child been attending kindergarten?

Month and year (e.g., September 2021): _______________________

5. Can your child understand and follow instructions in German?

☐ Yes ☐ No

6. Does your child have a congenital or acquired impairment?

☐ Yes ☐ No

If yes, please specify:

7. Regarding your child, how important are the following assessments to you?

(0 = not at all important, 5 = very important)

Annual general medical check-up:	☐ 0 ☐ 1 ☐ 2 ☐ 3 ☐ 4 ☐ 5
Dental health assessment:	☐ 0 ☐ 1 ☐ 2 ☐ 3 ☐ 4 ☐ 5
Assessment of motor skills:	☐ 0 ☐ 1 ☐ 2 ☐ 3 ☐ 4 ☐ 5
Eye examination:	☐ 0 ☐ 1 ☐ 2 ☐ 3 ☐ 4 ☐ 5
Assessment of language skills:	☐ 0 ☐ 1 ☐ 2 ☐ 3 ☐ 4 ☐ 5

© 2025 Scheiber B et al. *JAMA Network Open*.

8. Would you support the idea of offering an annual mobility screening (motor skills assessment) in kindergarten?

☐ Yes ☐ No

Could you briefly explain your reasoning?

____
____
____

9. Have you fully participated in the “mother-child pass” medical check-ups?

☐ Yes ☐ No

If no, up to what age did you participate in the check-ups?

☐ Up to age 1 ☐ Up to age 2 ☐ Up to age 3 ☐ Up to age 4 ☐ Up to age 5

10. Has your child ever been diagnosed with or shown signs of motor difficulties during a routine check-up?

☐ Yes ☐ No

If yes, what was identified?

____

Is this issue still present?

☐ Yes ☐ No

Thank you very much for completing this questionnaire and supporting your child’s participation in the mobility screening.

Please return the completed questionnaire along with the signed consent form in the provided envelope to your child’s kindergarten teacher by May 29, 2024.

© 2025 Scheiber B et al. *JAMA Network Open*.
