# Supplementary material for: Unrecognized Motor Difficulties and Developmental Coordination Disorder in Preschool Children
Source: JAMA Netw Open. 2025 Oct 7;8(10):e2536227. doi: 10.1001/jamanetworkopen.2025.36227 (PMC12505165; doi:10.1001/jamanetworkopen.2025.36227)
Supplement: Supplement 2. — Data Sharing Statement [file jamanetwopen-e2536227-s002.pdf]

Data Sharing Statement

Data
Data available: No

Additional Information
Explanation for why data not available: Data will be made available on request.
